# NF-κB activation and cytokine dysregulation: a pathogenic loop in breast cancer inflammation – a narrative review

**DOI:** 10.1097/MS9.0000000000004889

**Published:** 2026-04-20

**Authors:** Emmanuel Ifeanyi Obeagu

**Affiliations:** a Department of Biomedical and Laboratory Science, Africa University, Mutare, Zimbabwe; b Department of Molecular Medicine and Haematology, Faculty of Health Sciences, University of the Witwatersrand, Johannesburg, South Africa

**Keywords:** breast cancer, cytokine dysregulation, inflammation, NF-κB activation, tumor microenvironment

## Abstract

Chronic inflammation is a critical driver of breast cancer initiation and progression, with the nuclear factor kappa B (NF-κB) pathway acting as a central regulator of pro-inflammatory and oncogenic signaling. Dysregulated cytokine production reinforces NF-κB activation, establishing a self-perpetuating inflammatory loop that sustains tumor growth and immune evasion. This review explores the molecular interplay between NF-κB activation and cytokine dysregulation in breast cancer, emphasizing their combined role in shaping the tumor microenvironment and promoting malignancy. Persistent NF-κB activation – induced by cytokines such as interleukin-1β, interleukin-6, interleukin-8, and tumor necrosis factor-alpha – enhances transcription of genes governing proliferation, angiogenesis, and metastasis. In turn, these cytokines activate feedback loops that maintain NF-κB signaling, leading to chronic inflammation and therapy resistance. The NF-κB–cytokine axis also modulates immune checkpoint expression, supporting tumor immune escape. Understanding this dynamic interplay provides insight into novel therapeutic approaches, including NF-κB inhibitors, anti-cytokine antibodies, and combinatorial anti-inflammatory strategies. NF-κB activation and cytokine dysregulation form a pathogenic loop central to breast cancer inflammation, progression, and treatment resistance. Targeted disruption of this loop represents a promising avenue for precision-based therapeutic intervention and improved clinical outcomes.

## Introduction

Breast cancer remains the most prevalent malignancy among women worldwide, accounting for approximately one in four cancer diagnoses and one in six cancer-related deaths in women. While genetic and hormonal factors play significant roles in its pathogenesis, accumulating evidence underscores the crucial contribution of chronic inflammation to tumor initiation, progression, and metastasis. The inflammatory tumor microenvironment (TME) is not merely a bystander but an active participant in shaping the biological behavior of breast tumors, influencing their growth kinetics, immune interactions, and therapeutic responses^[^1^]^. Among the molecular mediators orchestrating this inflammatory milieu, nuclear factor kappa B (NF-κB) stands out as a master regulator. NF-κB is a transcription factor that controls the expression of genes involved in cell survival, proliferation, angiogenesis, and immune regulation. Under normal physiological conditions, NF-κB activation is transient and tightly controlled, allowing cells to respond to infection or injury. However, in breast cancer, NF-κB signaling becomes constitutively activated, leading to persistent expression of inflammatory and anti-apoptotic genes that promote malignant transformation^[^2^]^.

HIGHLIGHTS

- Nf-κb drives breast cancer inflammation via chronic activation and cytokine dysregulation.
- IL-1β, IL-6, IL-8, TNF-α, and TGF-β form a pathogenic feedback loop.
- Dysregulated cytokines promote EMT, angiogenesis, metastasis, and therapy resistance.
- Targeting NF-κB and cytokines offers promising therapeutic opportunities.
- Tumor microenvironment cross talk amplifies inflammation, suggesting biomarker-guided precision interventions.

Parallel to NF-κB activation, cytokine dysregulation plays a pivotal role in reinforcing inflammation and maintaining tumor-promoting signaling. Cytokines such as interleukin-1β (IL-1β), interleukin-6 (IL-6), interleukin-8 (IL-8), tumor necrosis factor-alpha (TNF-α), and transforming growth factor-beta (TGF-β) are frequently upregulated in breast cancer tissues and circulation. These cytokines not only mediate intercellular communication within the TME but also serve as powerful activators of NF-κB, forming a reciprocal feedback loop that sustains chronic inflammation. This NF-κB–cytokine circuit fuels oncogenic processes such as epithelial–mesenchymal transition (EMT), angiogenesis, metastasis, and immune evasion^[^3,4^]^. The pathogenic loop between NF-κB activation and cytokine imbalance represents a hallmark of inflammation-driven carcinogenesis. Pro-inflammatory cytokines such as IL-6 and TNF-α can activate the NF-κB pathway through IκB kinase (IKK)-mediated signaling, while NF-κB in turn induces the transcription of these same cytokines, perpetuating a self-sustaining inflammatory cycle. This continuous activation not only supports tumor cell proliferation and survival but also recruits immune and stromal cells that further amplify the inflammatory response. The end result is a microenvironment that favors tumor expansion, vascular remodeling, and resistance to both immune surveillance and conventional therapies^[^5,6^]^.

Furthermore, NF-κB activation is closely linked to hormone receptor signaling and oncogenic pathways in breast cancer. Estrogen receptor (ER)-negative and triple-negative breast cancers (TNBCs), in particular, exhibit elevated NF-κB activity and inflammatory cytokine expression, correlating with aggressive clinical behavior and poor prognosis. The persistent activation of NF-κB also contributes to chemotherapy and radiotherapy resistance, as it enhances DNA repair mechanisms, inhibits apoptosis, and promotes survival pathways within cancer cells^[^7,8^]^. Understanding this intricate network is critical for developing novel therapeutic strategies. Recent advances in molecular oncology have identified NF-κB–targeted therapies, anti-cytokine biologics, and natural anti-inflammatory compounds as promising interventions capable of disrupting this pathogenic feedback loop. However, given NF-κB’s essential role in normal immune homeostasis, achieving selective inhibition without compromising physiological immunity remains a major challenge^[^5,9,10^]^. This review delves into the molecular mechanisms linking NF-κB activation and cytokine dysregulation in breast cancer, elucidating how this interplay shapes the TME and contributes to tumor aggressiveness. It further discusses therapeutic strategies aimed at breaking this inflammatory circuit, highlighting emerging trends in precision medicine that could revolutionize the management of breast cancer through targeted modulation of inflammation.

## Aim

The aim of this narrative review is to comprehensively examine the interrelationship between NF-κB activation and cytokine dysregulation in the context of breast cancer inflammation. Specifically, it seeks to

- **Elucidate the molecular mechanisms** underlying NF-κB activation and its role in regulating inflammatory cytokine expression.

- **Analyze the bidirectional feedback loop** through which cytokines perpetuate NF-κB signaling, fostering a pro-tumorigenic microenvironment.
- **Highlight the clinical implications** of NF-κB–cytokine cross talk in breast cancer progression, metastasis, and therapeutic resistance.
- **Evaluate emerging therapeutic strategies** targeting the NF-κB–cytokine axis to disrupt inflammation-driven carcinogenesis.

Through this synthesis, the review aims to provide a clearer understanding of how targeted modulation of the NF-κB–cytokine loop can advance the development of precision anti-inflammatory therapies in breast cancer management.

## Methods

This narrative review was conducted through an extensive and integrative literature synthesis designed to explore the molecular and clinical interplay between NF-κB activation and cytokine dysregulation in breast cancer–related inflammation. A systematic search of published literature was performed across major biomedical databases including PubMed, Scopus, Web of Science, and Google Scholar. The search covered studies published between 2000 and 2025 to capture both foundational mechanisms and recent advancements. The following key terms and Boolean combinations were applied: “NF-κB activation,” “cytokine dysregulation,” “breast cancer inflammation,” “tumor microenvironment,” “pro-inflammatory cytokines,” “IL-6,” “TNF-α,” “TGF-β,” “IL-1β,” and “NF-κB inhibitors.” Reference lists of relevant articles were manually screened to identify additional studies of importance.

Eligible sources included original research articles, clinical studies, meta-analyses, systematic reviews, and mechanistic reports focusing on NF-κB signaling, cytokine expression, TME modulation, and inflammation-driven carcinogenesis in breast cancer. Studies conducted on both human tissues and experimental models (*in vitro* and *in vivo*) were included to ensure a comprehensive molecular-to-clinical perspective. Publications not available in English or lacking peer review were excluded to maintain data reliability.

The selected articles were analyzed thematically, with emphasis placed on

1. Mechanisms of NF-κB activation (canonical and non-canonical pathways).

2. Cytokine-mediated regulation and feedback activation of NF-κB.

3. The impact of this interaction on tumor progression, immune modulation, and metastasis.

4. Therapeutic interventions targeting the NF-κB–cytokine axis.

All findings were synthesized in a descriptive and interpretive manner, rather than quantitatively, in keeping with the principles of a narrative review. The goal was to integrate molecular insights with translational and clinical implications to provide a holistic understanding of the NF-κB–cytokine feedback loop as a pathogenic mechanism in breast cancer inflammation.

## NF-κB signaling pathways: canonical and non-canonical mechanisms

The NF-κB family constitutes a group of transcription factors that play a central role in regulating immune responses, inflammation, cellular survival, and oncogenesis. In breast cancer, the dysregulation of NF-κB signaling has emerged as a defining molecular event linking chronic inflammation to tumor development and progression. This signaling network operates through two major activation routes – the canonical and non-canonical pathways – each characterized by distinct molecular triggers, intermediates, and biological outcomes^[^11^]^. The canonical NF-κB pathway represents the classical and most extensively studied mechanism of activation. It is primarily triggered by pro-inflammatory stimuli such as TNF-α, IL-1β, lipopolysaccharides, and signals from pattern recognition receptors like Toll-like receptors^[^4^]^. In resting cells, NF-κB dimers – commonly the p65 (RelA)/p50 complex – remain sequestered in the cytoplasm by binding to inhibitory IκB proteins. Upon stimulation, these inhibitory proteins are phosphorylated by the IKK complex, composed of IKKα, IKKβ, and the regulatory subunit NEMO (IKKγ). Phosphorylation targets IκB for ubiquitination and subsequent proteasomal degradation, releasing NF-κB dimers to translocate into the nucleus^[^12^]^.

Once in the nucleus, NF-κB binds to κB enhancer elements within the DNA to induce transcription of genes involved in cell proliferation (cyclin D1), survival (Bcl-2, survivin), angiogenesis [vascular endothelial growth factor (VEGF)], and cytokine production (IL-6, IL-8, TNF-α). These cytokines, in turn, further activate NF-κB, establishing a positive feedback loop that sustains chronic inflammation^[^13,14^]^. In breast cancer, constitutive activation of the canonical NF-κB pathway has been associated with aggressive tumor phenotypes, particularly in TNBC. It drives EMT, enhances metastatic potential, and promotes resistance to apoptosis, thereby linking inflammation directly to oncogenic transformation^[^15^]^. In contrast, the non-canonical (alternative) NF-κB pathway is activated by a more limited set of stimuli, primarily those involved in lymphoid organogenesis and immune regulation, including CD40 ligand, B-cell activating factor, and lymphotoxin-β. This pathway is independent of IKKβ and NEMO, relying instead on the NF-κB–inducing kinase (NIK) and IKKα homodimers^[^16^]^.

Under normal conditions, NIK is maintained at low levels through continuous ubiquitin-mediated degradation. Activation signals stabilize NIK, leading to phosphorylation and activation of IKKα. IKKα subsequently induces the proteolytic processing of the NF-κB2 precursor protein p100 into p52, which forms heterodimers with RelB. The resulting p52/RelB complex translocates into the nucleus to regulate genes involved in cell differentiation, immune signaling, and tumor survival^[^17^]^. Although the non-canonical pathway is less studied in breast cancer than the canonical one, growing evidence suggests that it contributes to tumor growth, angiogenesis, and immune evasion. Aberrant NIK stabilization and p100 processing have been detected in ER-negative tumors, suggesting a link between this pathway and hormone-independent tumor progression. Moreover, cross talk between canonical and non-canonical NF-κB signaling allows for coordinated regulation of inflammatory gene expression, reinforcing tumor-promoting processes^[^18^]^.

The integration between the canonical and non-canonical pathways adds another layer of complexity to NF-κB biology in breast cancer. Both routes converge on shared target genes that regulate inflammation, cytokine production, and cellular stress responses. For instance, IL-6 and TNF-α produced through canonical activation can indirectly stimulate non-canonical signaling components, while RelB/p52 complexes modulate the duration and intensity of canonical responses^[^19^]^. This bidirectional communication ensures that NF-κB activation is not a transient event but a sustained process capable of reprogramming the TME. The resultant inflammatory state promotes angiogenesis, stromal remodeling, and immunosuppression – all of which enhance breast cancer aggressiveness and metastatic potential^[^20^]^.

Aberrant NF-κB activation in breast cancer has profound clinical implications. Its persistent activation correlates with poor prognosis, chemotherapy resistance, and increased metastatic burden. NF-κB activity also drives the secretion of pro-inflammatory cytokines that recruit tumor-associated macrophages (TAMs) and cancer-associated fibroblasts (CAFs), creating a vicious cycle of inflammation and immune suppression^[^21^]^. Targeting these signaling nodes – either by inhibiting IKK complexes, blocking cytokine receptors, or modulating NIK activity – has emerged as a potential strategy to disrupt the NF-κB–driven inflammatory circuit. Understanding the molecular intricacies of these pathways provides a foundation for designing precise and effective interventions that can halt inflammation-induced tumor progression in breast cancer^[^22^]^.

## Cytokine dysregulation and NF-κB feedback activation

Cytokines serve as crucial molecular messengers within the TME, orchestrating cellular communication between cancer cells, immune cells, and stromal components. In breast cancer, this communication becomes pathologically altered due to cytokine dysregulation, resulting in an environment that fosters tumor survival, angiogenesis, and immune escape. Among the various pathways regulating these cytokines, NF-κB plays a central role. The interaction between cytokine overproduction and NF-κB activation forms a self-perpetuating inflammatory feedback loop, which sustains oncogenic signaling and drives disease progression^[^23,24^]^. Breast cancer tissues and patient sera often exhibit elevated levels of pro-inflammatory cytokines, notably IL-1β, IL-6, IL-8, TNF-α, and TGF-β. These cytokines exert multifaceted effects that contribute to tumorigenesis. IL-1β and TNF-α initiate inflammatory cascades that activate NF-κB, while IL-6 and IL-8 sustain tumor cell proliferation, migration, and resistance to apoptosis. TGF-β, though initially a tumor suppressor in early carcinogenesis, assumes a pro-metastatic role during advanced disease by promoting EMT and immune evasion^[^25,26^]^.

This imbalance between pro- and anti-inflammatory cytokines reshapes the TME into a chronic inflammatory state, characterized by persistent immune cell recruitment, oxidative stress, and continuous activation of intracellular signaling networks. The abnormal cytokine milieu not only drives tumor cell proliferation but also facilitates the recruitment of TAMs, CAFs, and myeloid-derived suppressor cells (MDSCs), all of which further amplify inflammatory signaling through NF-κB-dependent pathways^[^27^]^. NF-κB acts as both a target and an effector of cytokine signaling, forming the core of the inflammatory feedback loop. Cytokines such as TNF-α and IL-1β activate the canonical NF-κB pathway via the IKK complex, leading to IκB degradation and nuclear translocation of NF-κB dimers. Once activated, NF-κB induces transcription of genes encoding these same cytokines, ensuring their sustained expression and secretion^[^28^]^.

This reciprocal activation forms a self-amplifying cycle: cytokines stimulate NF-κB, and NF-κB, in turn, enhances cytokine production. The result is a persistent state of pathological inflammation, which not only supports tumor cell proliferation but also protects cancer cells from immune-mediated destruction and apoptosis. For instance, TNF-α stimulation of NF-κB upregulates anti-apoptotic genes such as *Bcl-2, Bcl-xL*, and *survivin*, promoting tumor cell longevity^[^29^]^. Additionally, NF-κB-driven transcription of IL-6 and IL-8 enhances activation of the JAK/STAT3 and MAPK pathways, which cooperate with NF-κB to further promote survival and angiogenesis. This synergistic interplay between cytokine signaling and NF-κB activation effectively reprograms breast cancer cells toward a more aggressive and therapy-resistant phenotype^[^30^]^. The functional outcome of NF-κB-mediated cytokine dysregulation is a sustained inflammatory microenvironment conducive to cancer progression. Elevated IL-6 levels correlate with advanced tumor grade, ER-negative status, and reduced overall survival. IL-8, another NF-κB target, promotes endothelial cell migration and neovascularization through upregulation of VEGF, supporting tumor angiogenesis^[^31^]^.

TGF-β plays a dual role within this feedback loop. Under NF-κB influence, TGF-β signaling transitions from tumor-suppressive to tumor-promoting, facilitating EMT, invasiveness, and metastatic dissemination. Moreover, chronic NF-κB activation induces immunosuppressive cytokines that inhibit cytotoxic T-cell function while expanding regulatory T cells (Tregs), further enhancing immune evasion^[^32^]^. In this context, NF-κB and cytokine dysregulation act as co-conspirators that continuously remodel the tumor landscape. The result is a vicious inflammatory cycle that supports angiogenesis, increases matrix degradation, enhances cell motility, and protects cancer cells from chemotherapy- or radiation-induced apoptosis^[^33^]^. The NF-κB–cytokine feedback system extends beyond tumor cells to the surrounding stromal and immune compartments. TAMs, for example, release TNF-α and IL-6, which further activate NF-κB in tumor cells. In return, cancer cells secrete colony-stimulating factors (CSFs) and chemokines (e.g., CCL2, CXCL12) that recruit more macrophages, creating a feed-forward inflammatory loop^[^34^]^.

Similarly, CAFs respond to NF-κB-driven cytokines by producing matrix metalloproteinases (MMPs) that degrade extracellular matrix (ECM) components, paving the way for invasion and metastasis. MDSCs also exploit this cytokine milieu to inhibit T-cell activity, ensuring immune suppression within the TME. This multifaceted cross talk underscores how cytokine dysregulation, under the governance of NF-κB, transforms the breast cancer microenvironment into a hub of chronic inflammation and immune tolerance^[^35^]^. Clinically, high circulating levels of IL-6, IL-8, and TNF-α have been associated with poor prognosis, increased metastatic potential, and resistance to systemic therapies in patients with breast cancer. These cytokines not only serve as biomarkers of disease aggressiveness but also represent potential therapeutic targets. Inhibiting cytokine-NF-κB feedback has shown promise in preclinical models, where agents targeting IL-6R (e.g., tocilizumab), TNF-α (e.g., infliximab), or IKKβ suppress tumor growth and enhance sensitivity to chemotherapy (Table 1)^[^25^]^.

**Table 1:** Key pro-inflammatory cytokines in breast cancer and their NF-κB-mediated effects.

Cytokine	Source in TME	NF-κB role	Pathogenic effects in breast cancer	Clinical relevance
IL-1β	Tumor cells, TAMs	Activates canonical NF-κB pathway	Promotes inflammation, EMT, angiogenesis	Associated with aggressive tumors, poor prognosis
IL-6	Tumor cells, CAFs, TAMs	NF-κB-dependent transcription	Stimulates proliferation, survival, therapy resistance	Biomarker for metastasis and chemoresistance
IL-8 (CXCL8)	Tumor cells, CAFs	NF-κB target	Enhances angiogenesis, migration, invasion	Correlates with metastatic potential
TNF-α	TAMs, tumor cells	Canonical NF-κB activator	Induces pro-survival genes, chronic inflammation	Linked to poor prognosis and therapy resistance
TGF-β	CAFs, tumor cells	Modulated by NF-κB	Facilitates EMT, immune evasion, metastasis	Higher expression in aggressive subtypes

## Molecular cross talk in the TME

The TME in breast cancer is a complex ecosystem composed of malignant epithelial cells, immune infiltrates, stromal fibroblasts, endothelial cells, and ECM components. Within this milieu, NF-κB activation and cytokine dysregulation orchestrate a dynamic molecular cross talk that drives tumor progression, metastasis, and immune evasion. Rather than acting in isolation, cancer cells leverage the surrounding stromal and immune compartments to create a self-reinforcing pro-tumorigenic network^[^36^]^. TAMs represent a major component of the breast cancer TME, often adopting an M2-like, pro-tumor phenotype. TAMs secrete cytokines such as TNF-α, IL-6, IL-8, and chemokines like CCL2, which activate NF-κB in both tumor and stromal cells. In turn, NF-κB activation in cancer cells enhances the production of CSFs and chemokines, further recruiting TAMs. This bidirectional communication establishes a feed-forward loop that promotes angiogenesis, matrix remodeling, and immune suppression^[^37^]^.

Furthermore, TAM-derived cytokines contribute to therapy resistance by upregulating anti-apoptotic proteins via NF-κB, protecting tumor cells from chemotherapeutic-induced cell death. Elevated TAM density and associated NF-κB-driven cytokine expression correlate with aggressive breast cancer phenotypes and poor clinical outcomes, particularly in triple-negative and HER2-positive tumors^[^38^]^. CAFs are activated stromal cells that play a pivotal role in shaping the TME. Through NF-κB-mediated signaling, tumor cells induce CAFs to secrete MMPs, fibronectin, and collagen-modifying enzymes, which remodel the ECM to facilitate tumor invasion and metastasis. Additionally, CAFs produce cytokines such as IL-6 and TGF-β, which further activate NF-κB in cancer cells, reinforcing EMT and invasiveness. This reciprocal interaction between CAFs and tumor cells exemplifies how the NF-κB–cytokine loop extends beyond individual cancer cells to the broader stromal compartment^[^39^]^.

Angiogenesis, a hallmark of breast cancer progression, is also tightly regulated by NF-κB-cytokine cross talk. NF-κB-induced secretion of VEGF, IL-8, and TNF-α from tumor cells and stromal components stimulates endothelial cell proliferation, migration, and tube formation. The resultant neovasculature not only supports tumor growth through enhanced nutrient and oxygen delivery but also provides routes for metastatic dissemination. Moreover, inflammatory cytokines within the TME increase endothelial permeability, facilitating tumor cell intravasation and extravasation^[^40^]^. Beyond structural support, the TME is populated by immune cells whose functions are co-opted by the NF-κB–cytokine axis. Tregs, MDSCs, and tolerogenic dendritic cells are recruited by NF-κB-driven chemokines and cytokines, establishing an immunosuppressive microenvironment. This network suppresses cytotoxic T-cell activity and natural killer cell function, enabling immune escape and tumor survival. Concurrently, NF-κB activation in tumor and stromal cells amplifies the expression of PD-L1, IDO, and other checkpoint molecules, further dampening anti-tumor immunity^[^41^]^. The integration of signals between tumor cells, immune infiltrates, and stromal components creates a self-sustaining pathogenic loop. NF-κB-driven cytokine production coordinates angiogenesis, matrix remodeling, and immune suppression simultaneously, creating a TME optimized for tumor growth, invasion, and resistance to therapy. The TME thus acts as a dynamic amplifier of NF-κB signaling and cytokine dysregulation, highlighting the necessity of therapeutic approaches that target both cancer cells and their microenvironmental collaborators^[^42^]^.

## Therapeutic targeting of the NF-κB–cytokine axis

The persistent activation of NF-κB and the accompanying cytokine dysregulation in breast cancer establish a self-sustaining pathogenic loop, making this axis a compelling therapeutic target. Given its central role in promoting tumor survival, angiogenesis, metastasis, and immune evasion, disrupting NF-κB signaling or modulating cytokine activity has emerged as a strategic approach to mitigate inflammation-driven tumor progression^[^8^]^. Direct pharmacological inhibition of NF-κB has been explored through multiple mechanisms. Small-molecule inhibitors targeting IKK complexes, such as BMS-345541 and MLN120B, prevent IκB phosphorylation, thereby sequestering NF-κB in the cytoplasm and suppressing its transcriptional activity. Proteasome inhibitors, notably bortezomib, indirectly inhibit NF-κB by preventing degradation of IκB, resulting in reduced NF-κB nuclear translocation. Preclinical studies demonstrate that these agents reduce proliferation, induce apoptosis, and sensitize breast cancer cells to chemotherapy. However, systemic NF-κB inhibition carries the risk of immune suppression, highlighting the need for tumor-selective strategies^[^43^]^.

Targeting specific pro-inflammatory cytokines represents another effective approach to disrupting the NF-κB–cytokine feedback loop. IL-6 receptor antagonists such as tocilizumab and siltuximab inhibit the IL-6–JAK/STAT3 axis, reducing NF-κB activation and attenuating tumor-promoting inflammation. Similarly, TNF-α inhibitors like infliximab and etanercept prevent canonical NF-κB pathway activation. Preclinical breast cancer models have shown that anti-cytokine therapy decreases angiogenesis, EMT, and metastatic potential. Importantly, these therapies may also enhance the efficacy of chemotherapy and radiotherapy by reducing NF-κB-mediated resistance mechanisms^[^44^]^. Given the redundancy and plasticity of inflammatory signaling, combination therapies targeting both NF-κB and cytokine pathways offer a promising strategy. For example, co-inhibition of NF-κB and IL-6 or IL-8 can simultaneously block tumor proliferation, angiogenesis, and immune evasion. Additionally, integrating immune checkpoint inhibitors with NF-κB or cytokine-targeted therapies can restore anti-tumor immunity, particularly in immunologically “cold” tumors characterized by chronic NF-κB-driven inflammation^[^45^]^.

Several natural and bioactive compounds have demonstrated NF-κB and cytokine-modulating properties. Curcumin, resveratrol, epigallocatechin-3-gallate (EGCG), and quercetin inhibit NF-κB activation and reduce pro-inflammatory cytokine production in breast cancer models. These agents offer potential adjunctive benefits due to their low toxicity and anti-inflammatory properties, though clinical translation requires rigorous testing. Emerging nanoparticle-based delivery systems are also being explored to selectively target NF-κB signaling within tumor tissues, minimizing systemic immunosuppression^[^46,47^]^. Therapeutic targeting of the NF-κB–cytokine axis must balance efficacy with safety, as NF-κB is essential for normal immune function. Selective inhibition strategies, including tumor-specific delivery, context-dependent pathway modulation, and transient blockade, are under investigation to mitigate adverse effects. Furthermore, patient stratification using NF-κB activity levels, cytokine profiles, and inflammatory signatures may optimize treatment outcomes and identify individuals most likely to benefit from these targeted interventions^[^47^]^. Future research should focus on the integration of multi-targeted therapies, combining NF-κB inhibitors, cytokine blockers, and immunomodulatory agents to disrupt the pathogenic feedback loop more effectively. Additionally, elucidating the interplay between NF-κB signaling, cytokine networks, and hormone receptor pathways may inform precision-based approaches for specific breast cancer subtypes, particularly triple-negative and hormone receptor–negative tumors. Advances in biomarker-guided therapy and TME modulation hold promise for translating these molecular insights into clinically meaningful outcomes (Table 2)^[^48^]^.

**Table 2:** Therapeutic strategies targeting NF-κB–cytokine axis in breast cancer.

Therapeutic approach	Mechanism of action	Target	Preclinical/Clinical evidence	Potential benefits
IKK/NF-κB inhibitors (e.g., BMS-345541)	Blocks IκB phosphorylation, prevents NF-κB nuclear translocation	IKK complex	Reduces proliferation, induces apoptosis in preclinical models	Suppresses tumor growth and inflammatory signaling
Proteasome inhibitors (e.g., bortezomib)	Prevents IκB degradation	Proteasome/NF-κB	Reduces NF-κB activity, sensitizes tumor cells to chemo	Enhances therapy response, induces apoptosis
Anti-IL-6 therapy (tocilizumab, siltuximab)	Blocks IL-6/IL-6R signaling, reduces NF-κB feedback	IL-6/IL-6R	Decreases tumor growth and angiogenesis in preclinical models	Attenuates inflammation-driven progression
Anti-TNF-α therapy (infliximab, etanercept)	Neutralizes TNF-α, prevents canonical NF-κB activation	TNF-α	Reduces metastasis and inflammatory signaling in studies	Modulates microenvironment, improves therapy sensitivity
Natural compounds (curcumin, resveratrol, EGCG)	Inhibit NF-κB activation, reduce cytokine production	NF-κB, multiple cytokines	Preclinical evidence of anti-proliferative effects	Low toxicity, adjunctive therapy potential
Combination therapy	Concurrent NF-κB and cytokine blockade	NF-κB + cytokines	Preclinical models show synergy	Disrupts feedback loop, reduces immune suppression, inhibits metastasis

## Clinical implications

The interplay between NF-κB activation and cytokine dysregulation has profound clinical relevance in breast cancer, influencing prognosis, therapeutic response, and disease progression. Elevated NF-κB activity and persistent pro-inflammatory cytokine signaling correlate with aggressive tumor phenotypes, including triple-negative and HER2-positive breast cancers, which are often resistant to conventional therapies. Chronic NF-κB-mediated inflammation promotes EMT, angiogenesis, and metastatic dissemination, thereby increasing the likelihood of recurrence and poor clinical outcomes^[^49^]^. From a diagnostic perspective, measuring circulating levels of IL-6, IL-8, TNF-α, and other NF-κB-inducible cytokines may serve as prognostic biomarkers. High cytokine concentrations can identify patients at greater risk of metastasis, chemoresistance, or immune evasion, guiding personalized treatment strategies. NF-κB activity assays in tumor biopsies may also inform subtype-specific therapeutic interventions, particularly in aggressive or hormone receptor–negative cancers^[^50^]^.

Therapeutically, targeting the NF-κB–cytokine axis provides multiple clinical opportunities. Direct NF-κB inhibitors, anti-cytokine antibodies, and combination strategies have the potential to sensitize tumors to chemotherapy, reduce metastatic spread, and restore anti-tumor immunity. Integrating these approaches with immune checkpoint inhibitors or hormone-targeted therapies could further enhance clinical efficacy, especially in patients with an inflamed TME^[^22^]^. Moreover, understanding NF-κB-mediated cytokine dysregulation can aid in stratifying patients for clinical trials, identifying those most likely to benefit from inflammation-targeted therapies. Monitoring treatment response through cytokine profiling may allow early detection of therapy resistance and enable timely adjustments in therapeutic regimens.

## Future directions

Emerging insights into the NF-κB–cytokine axis in breast cancer highlight several avenues for translational research and therapeutic innovation. Future studies should prioritize precision-targeted approaches that disrupt the pathogenic loop without compromising physiological immune function. One promising direction involves the development of tumor-selective NF-κB inhibitors, including nanoparticle-based delivery systems and context-specific small molecules, to minimize systemic toxicity while effectively suppressing pro-tumorigenic signaling^[^25^]^. Parallel strategies should focus on multi-cytokine blockade, targeting IL-6, IL-8, TNF-α, and TGF-β concurrently to overcome redundancy within inflammatory networks. Integrating these interventions with immune checkpoint inhibitors or adoptive cell therapies could restore anti-tumor immunity and enhance clinical response in immunologically “cold” breast cancers^[^51^]^.

Biomarker-guided patient stratification represents another critical avenue. Profiling NF-κB activation status, cytokine expression patterns, and inflammatory signatures in tumor biopsies and peripheral blood may identify patients most likely to benefit from targeted interventions, enabling personalized therapy. Longitudinal monitoring of these biomarkers could also facilitate early detection of therapeutic resistance, permitting timely regimen adjustments^[^52^]^. Further research is warranted to elucidate cross talk between NF-κB, cytokines, and hormone receptor signaling, particularly in triple-negative and ER-negative tumors. Understanding how these pathways converge to promote EMT, metastasis, and therapy resistance may uncover novel combinatorial targets^[^7,53^]^. Preclinical models that better recapitulate the TME, including organoids and humanized mouse models, will be essential to evaluate the efficacy and safety of emerging NF-κB–cytokine-directed therapies. Collaborative clinical trials integrating molecular insights, biomarker-based stratification, and combination therapeutics hold the potential to translate these mechanistic findings into improved patient outcomes^[^54^]^.

## Conclusion

The NF-κB–cytokine axis represents a central pathogenic loop in breast cancer, linking chronic inflammation to tumor initiation, progression, metastasis, and therapy resistance. Dysregulated cytokine production, including IL-1β, IL-6, IL-8, TNF-α, and TGF-β, drives persistent NF-κB activation, creating a self-perpetuating inflammatory microenvironment that supports malignant behavior and immune evasion. Understanding the molecular intricacies of this loop provides a foundation for therapeutic intervention, with strategies ranging from direct NF-κB inhibition to anti-cytokine therapies and combination approaches that integrate immunomodulation. These interventions have the potential to disrupt inflammation-driven tumor progression, enhance sensitivity to conventional therapies, and improve clinical outcomes.

Future research should focus on tumor-specific targeting, biomarker-guided patient stratification, and multi-modal treatment strategies to optimize clinical efficacy while minimizing systemic immune suppression. By translating mechanistic insights into precision medicine approaches, targeting the NF-κB–cytokine feedback loop offers a promising avenue to combat aggressive breast cancer subtypes and reduce disease recurrence.
